# Anemia and Blood Transfusion in Patients with Isolated Traumatic Brain Injury

**DOI:** 10.1155/2015/672639

**Published:** 2015-10-28

**Authors:** Hasan M. Al-Dorzi, Waleed Al-Humaid, Hani M. Tamim, Samir Haddad, Ahmad Aljabbary, Abdulaziz Arifi, Yaseen M. Arabi

**Affiliations:** ^1^Intensive Care Department, King Abdulaziz Medical City, Riyadh 11426, Saudi Arabia; ^2^College of Medicine, King Saud Bin Abdulaziz University for Health Sciences, Riyadh 11426, Saudi Arabia; ^3^Epidemiology and Biostatistics, King Abdullah International Medical Research Center, King Saud Bin Abdulaziz University for Health Sciences, Riyadh 11426, Saudi Arabia; ^4^Department of Internal Medicine, American University of Beirut Medical Center, Beirut 1107 2020, Lebanon; ^5^Neurosurgery/Surgery Department, King Abdulaziz Medical City, Riyadh 11426, Saudi Arabia

## Abstract

*Rationale*. By reducing cerebral oxygen delivery, anemia may aggravate traumatic brain injury (TBI) secondary insult. This study evaluated the impact of anemia and blood transfusion on TBI outcomes. *Methods*. This was a retrospective cohort study of adult patients with isolated TBI at a tertiary-care intensive care unit from 1/1/2000 to 31/12/2011. Daily hemoglobin level and packed red blood cell (PRBC) transfusion were recorded. Patients with hemoglobin < 10 g/dL during ICU stay (anemic group) were compared with other patients. *Results*. Anemia was present on admission in two (2%) patients and developed in 48% during the first week with hemoglobin < 7 g/dL occurring in 3.0%. Anemic patients had higher admission Injury Severity Score and underwent more craniotomy (50% versus 13%, *p* < 0.001). Forty percent of them received PRBC transfusion (2.8 ± 1.5 units per patient, median pretransfusion hemoglobin = 8.8 g/dL). Higher hospital mortality was associated with anemia (25% versus 6% for nonanemic patients, *p* = 0.01) and PRBC transfusion (38% versus 9% for nontransfused patients, *p* = 0.003). On multivariate analysis, only PRBC transfusion independently predicted hospital mortality (odds ratio: 6.8; 95% confidence interval: 1.1–42.3). *Conclusions*. Anemia occurred frequently after isolated TBI, but only PRBC transfusion independently predicted mortality.

## 1. Introduction

Traumatic brain injury (TBI) is a leading cause of disability and death worldwide [1]. Head trauma causes primary brain injury, which is induced by the mechanical damage from the trauma itself. This may be followed by secondary brain injury that evolves over time and is caused by several mechanisms such as increased intracranial pressure and decreased oxygen delivery to the brain [2–4]. As serum hemoglobin is a major determinant of oxygen delivery, anemia may hypothetically lead to worse outcomes in TBI patients.

Anemia is common in TBI patients but has not been carefully described [5]. One study found that 40–50% of TBI patients had at least one hematocrit < 30% during their intensive care unit (ICU) stay [5]. Another study observed that hemoglobin < 8.0 g/dL occurred in 22% of severe TBI patients [6]. Studies that evaluated the relationship between anemia and TBI outcomes found conflicting results but generally had important methodological weaknesses. A retrospective single-center study of 133 patients with severe TBI admitted between 1998 and 2002 and managed according to the European Brain Injury Consortium guidelines found that hemoglobin < 8.0 g/dL did not predict adverse outcomes such as in-hospital mortality and Glasgow Outcome Scale at six months [6]. Predictors of unfavorable outcome were older age, lower Glasgow Coma Scale (GCS), hypotension during the first treatment day, elevated blood sugar, and low serum albumin [6]. On the other hand, another retrospective study of 1150 TBI patients admitted between 1998 and 2005 found that anemia was a significant risk factor for mortality (adjusted odds ratio (OR): 1.59; 95% confidence interval (CI): 1.13–2.24) [7]. Blood transfusion would theoretically abate the effects of anemia on oxygen delivery and so clinicians may use it to prevent secondary brain injury. Clinical studies on the relationship between blood transfusion and TBI outcomes also found conflicting results. One study showed that blood transfusion was associated with worse neurologic outcomes at discharge [8], but another did not [9]. Other studies found increased complications such as deep vein thrombosis with packed red blood cells (PRBCs) transfusion and even mortality [10].

Because of the conflicting evidence, anemia management in TBI patients remains controversial [5]. The objectives of this study were to determine anemia incidence in patients with isolated TBI during ICU stay, describe blood transfusion practices in them, and assess the impact of anemia and blood transfusion on their outcomes.

## 2. Methods

This was a retrospective cohort study of adult patients with isolated TBI from January 2000 to December 2011 in the adult ICU at King Abdulaziz Medical City, Riyadh, Saudi Arabia. King Abdulaziz Medical City was a 900-bed tertiary-care Joint Commission International-accredited academic center. The ICU was a 21-bed medical-surgical unit, staffed by on-site board certified critical care physicians. Approximately, 900 patients were admitted to the ICU annually, with close to 25% of admissions being trauma cases. All patients with isolated TBI admitted to this ICU within the study period were included. Severe TBI patients were managed with a head injury protocol, which was derived from the guidelines published by the Brain Trauma Foundation and required maintaining the following for seven days: euvolemia, mean arterial pressure ≥ 80 mmHg when intracranial pressure monitoring was not available, cerebral perfusion pressure > 60 mmHg when intracranial pressure monitoring was available, PaO_2_ > 80 mmHg, and PaCO_2_ from 35 to 40 mmHg [11]. This protocol was shown to be associated with reduced hospital mortality [12]. The target mean arterial pressure for patients not on the protocol was ≥65 mmHg. Exclusion criteria included age < 14 years (age cutoff for pediatric service admission in our hospital), significant injury of other organs, bleeding at other sites, penetrating head trauma, patients with chronic anemia, and death or brain death during the first 24 hours after TBI. The Institutional Review Board of King Abdulaziz Medical City approved the study.

In our ICU, hemoglobin level is routinely checked every morning. In case of a significant drop in hemoglobin level or an acute change in hemodynamics and after transfusion, the level would be repeated with the frequency determined by the treating intensivist. In this study, admission and daily blood hemoglobin levels during the first 7 days of ICU stay were recorded. According to the World Health Organization, anemia is usually defined as hemoglobin < 13.0 g/dL in men > 15 years old and <12.0 g/dL in nonpregnant women > 15 years old [13]. As hemoglobin < 10.0 g/dL is believed to be a more clinically relevant cutoff than <12 g/dL and is frequently used to determine the need for blood transfusion [5, 14, 15], we divided patients into an anemic group when their nadir hemoglobin was <10.0 g/dL and a nonanemic group (nadir hemoglobin ≥ 10.0 g/dL). Anemia was further categorized as moderate (hemoglobin 7.0–9.9 g/dL) and severe (hemoglobin < 7.0 g/dL, the threshold for restrictive blood transfusion [15]). Other collected information included age, gender, Acute Physiology and Chronic Health Evaluation (APACHE) II score [16], Injury Severity Score, admission GCS score, type of head injury based on the review of brain computed tomography, previous history of anemia such as sickle cell anemia or iron deficiency anemia, the ratio of partial pressure of oxygen to the fraction of inspired oxygen (PaO_2_ : FiO_2_), requirement of mechanical ventilation, requirement of craniotomy performed to evacuate bleeding and/or insert external ventricular drain, typically done before ICU admission, daily mean arterial pressure, daily mean intracranial pressure, daily mean cerebral perfusion pressure, management with head injury protocol, and pretransfusion hemoglobin. In addition, PRBC, platelet, and fresh frozen plasma transfusions were recorded. Outcome measures included the development of venous thromboembolism, ventilator-associated pneumonia, seizures, duration of mechanical ventilation, tracheostomy, length of stay in the ICU and hospital, and ICU and hospital mortality.

### 2.1. Statistical Analysis

All statistical analyses were conducted using Statistical Analysis System (SAS, version 9.0; SAS Institute, Cary, NC). Quantitative variables were presented as means with standard deviation (SD) or as median with the first and third quartiles. Qualitative variables were presented as frequencies with percentages. Chi Square, Fisher's exact, and Student's *t*-tests were used to compare groups as appropriate. The prevalence of anemia on admission (based on admission hemoglobin) and its incidence in the first seven days of ICU stay (based on the lowest and mean hemoglobin readings) were calculated. The main comparisons were made between patients who had anemia < 10.0 g/dL during the first seven days of ICU stay and those whose hemoglobin remained ≥10.0 g/dL. Multivariate logistic regression analysis was used to assess the relationship between anemia (hemoglobin < 10.0 g/dL), PRBC transfusion, and hospital mortality in all patients and in patients with severe TBI (GCS score ≤ 8). As anemia and blood transfusion are related to each other, the variance inflation factor between these two variables was calculated and was <2, indicating no multicollinearity. Hence, both variables were entered in the same model. Additional variables entered in the model were a priori chosen as those that are significantly different between the anemia groups in addition to the severity of illness and so included age, admission GCS score, APACHE II score, Injury Severity Score, requirement for craniotomy, and use of head injury protocol. Results were presented as OR with 95% CI. A *p* value < 0.05 was considered significant. The available sample size allowed the detection of an association between anemia and death with an OR of 6.4.

## 3. Results

### 3.1. Baseline Characteristics

During the study period, 101 patients were admitted to the ICU with isolated TBI. The baseline characteristics of patients are described in Table 1. Most (80.2%) patients had severe TBI with GCS score ≤ 8 on admission. Subarachnoid hemorrhage was the most common type of injury and occurred in 28 (28%) patients, followed by subdural hematoma (25%). Thirty-one (31%) patients had craniotomy. Most patients required mechanical ventilation (97%) and were treated with head injury protocol (75%).

### 3.2. Prevalence of Anemia on Admission and Incidence during Stay

**Figure 1:**
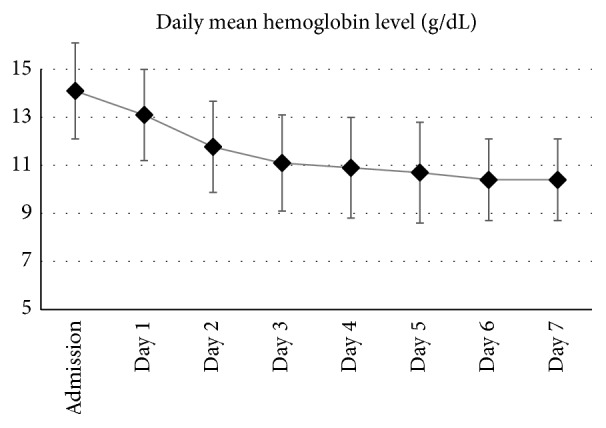
Course of hemoglobin in the first seven days of stay in the intensive care unit for patients with isolated traumatic brain injury. The error bars represent the standard deviation.

**Figure 2:**
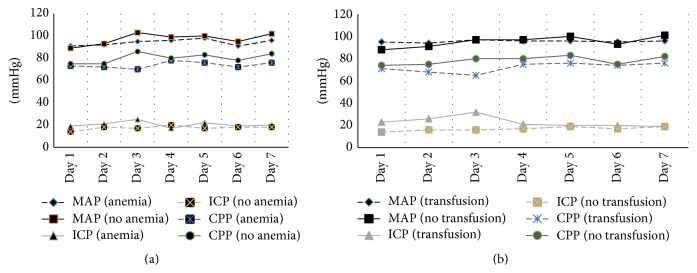
Mean arterial pressure (MAP), intracranial pressure (ICP) and cerebral perfusion pressure (CPP) in the first 7 days of stay in the intensive care unit. (a) Pressure values in patients who developed anemia (hemoglobin < 10 g/dL) and those who did not. (b) Pressure values in patients who received blood transfusion and those who did not. *p* values between groups were >0.05 for all points except for MAP on day 1 for transfused versus nontransfused patients (*p* = 0.04).

According to the World Health Organization definition, anemia was present in 26 (25.7%) patients on admission with hemoglobin < 10.0 g/dL present in only two patients.  Figure 1 describes the evolution of hemoglobin levels during the first 7 days of ICU stay. Nadir hemoglobin < 12.0 g/dL occurred in 19 (18.8%) patients during the first 7 days of ICU stay, while hemoglobin < 10.0 g/dL developed in 48%. Hemoglobin level dropped to 7.0–9.9 g/dL in 43 (42.6%) patients and to <7.0 g/dL in 4 (3.9%). Table 1 also describes the characteristics of patients according to the presence or absence of anemia (<10.0 g/dL). Compared with nonanemic patients, patients who had anemia during ICU stay were older (32 ± 16 versus 26 ± 12 years, *p* = 0.04) with higher Injury Severity Score but no difference in admission GCS score or APACHE II scores. Subdural hematoma and brain contusions were more common in anemic patients. More patients who developed anemia had craniotomy before ICU admission (50% versus 13%, *p* < 0.001) and management with head injury protocol during ICU stay (88% versus 64%, *p* = 0.01). The PaO_2_ : FiO_2_ ratio on admission and the mean arterial, intracranial, and cerebral perfusion pressure in the first 7 days of ICU stay were similar in the anemic and nonanemic groups (*p* > 0.05) as shown in Figure 2(a).

### 3.3. Transfusion of Blood Products

Obviously, patients with anemia (hemoglobin < 10.0 g/dL) were more likely to receive PRBC transfusion (40% versus 4%, *p* < 0.01). The median pretransfusion hemoglobin was 8.8 g/dL (Q1–Q3: 7.4–11.0 g/dL). Transfused patients received a median of 2 units of PRBCs (Q1–Q3: 2–3.5 units). Fifteen patients received fresh frozen plasma (median 2 units of FFP) (Q1–Q3: 1–3 units). Only two patients received platelet transfusion during ICU stay. The PaO_2_ :  FiO_2_ ratio on admission and the mean arterial, intracranial, and cerebral perfusion pressure in the first 7 days of ICU stay were similar in the transfused patients compared with those who did not receive PRBC transfusion (*p* > 0.05) except for higher mean arterial pressure of the first day in the transfused patients (95 ± 5 versus 88 ± 8 mmHg, *p* = 0.04) (Figure 2(b)). None of the patients received recombinant human erythropoietin.

### 3.4. Outcomes of Patients

Table 2 describes the outcomes of all patients and according to the presence or absence of anemia (hemoglobin < 10.0 g/dL). Venous thromboembolism including pulmonary embolism and deep venous thrombosis occurred in 8% of patients who developed anemia during stay compared with 2% in the nonanemic group; however, the differences were not statistically significant. The incidences of seizures and ventilator-associated pneumonia were similar in both groups. Tracheostomy was performed in 42% of the anemic patients and 32% of the nonanemic patients (*p* = 0.32). Patients who received PRBC transfusion had similar rates of venous thromboembolism, seizures, ventilator-associated pneumonia, and tracheostomy compared with nontransfused patients. Mechanical ventilation duration and ICU and hospital length of stay were also similar in anemic and nonanemic patients and in transfused and nontransfused patients.

Compared with other patients, anemic patients had higher ICU (23% versus 6%, *p* = 0.01) and hospital (25% versus 6%, *p* = 0.01) mortality. Hospital mortality was 0% in patients who did not develop anemia, 9% when hemoglobin fell to 10–11.9 g/dL during stay, 23% when hemoglobin fell to 7.0–9.9 g/dL, and 50% in patients who had hemoglobin below 7 g/dL (*p* = 0.02). Having hemoglobin level < 8 g/dL on ≥2 days was associated with higher mortality compared with <2 days (66.7% versus 15.4%; *p* = 0.004). PRBC transfusion was associated with higher ICU (38.1% versus 7.5% for nontransfused patients, *p* = 0.001) and hospital (38% versus 9% for nontransfused patients, *p* = 0.003) mortality. Patients receiving PRBC transfusion with pretransfusion hemoglobin < 8.0 g/dL had 22.2% mortality compared with 50% for patients with pretransfusion hemoglobin ≥ 8.0 g/dL (*p* = 0.37). On multivariate analysis, PRBC transfusion (OR: 6.8; 95% CI: 1.1–42.3), but not anemia (OR: 2.5; 95% CI: 0.4–15.8), was associated with hospital mortality. These associations persisted in patients with GCS score ≤ 8 (OR: 6.9; 95% CI: 1.1–42.6 for PRBC transfusion and OR: 2.4; 95% CI: 0.4–15.4 for anemia). The other predictors of hospital mortality for all patients were age (OR: 1.1 per year increment; 95% CI: 1.0–1.1), admission GCS score (OR: 0.6 per point increment; 95% CI: 0.4–0.9), and APACHE II score (OR: 1.2 per unit increment; 95% CI: 1.1–1.5).

## 4. Discussion

The main findings of this study were that anemia was relatively rare in patients with isolated TBI on hospital admission but became very common during ICU stay; anemia and PRBC transfusion were associated with higher mortality, but on multivariate analysis PRBC transfusion and not anemia was an independent predictor of mortality.

Trauma patients may become anemic initially when they present to the hospital due to blood loss from injuries. Although isolated TBI patients are expected to have less blood loss than patients with other penetrating injuries, the literature shows that anemia is common in blunt TBI [5, 6]. Schirmer-Mikalsen et al. observed that anemia (hemoglobin level < 8.0 g/dL) occurred in 22% of TBI patients [6]. Utter et al. found that around 40–50% of TBI patients have at least one hematocrit < 30% during their ICU stay [5]. Salim et al. showed that anemia was a significant risk factor for mortality (adjusted OR: 1.59; 95% CI: 1.13–2.24; *p* = 0.007) and for complications (adjusted OR: 1.95; 95% CI: 1.42–2.70; *p* < 0.0001) [7]. Using cutoff hemoglobin < 10.0 g/dL, our study showed that only two patients were anemic based on the admission hemoglobin. However, the hemoglobin level dropped in many patients, so that 48% of patients had hemoglobin < 10.0 g/dL during ICU stay. This is likely due to blood loss from procedures and phlebotomy for diagnostic blood tests [17] as well as bone marrow suppression due to critical illness [18]. As more patients who developed anemia had prior craniotomy, it is possible that the blood loss associated with this procedure contributed to the development of anemia. Only a minority (3%) of patients developed severe anemia (hemoglobin < 7.0 g/dL) and 42.6% developed moderate anemia (hemoglobin from 7.0 to 9.9 g/dL).

PRBC transfusion is commonly used in the treatment of anemia. For a long period, hemoglobin of 10.0 g/dL was the threshold for transfusion [17]. The critical care practice changed more than a decade ago as liberal PRBC transfusion was not shown to be beneficial in general ICU patients [15]. However, liberal transfusion in TBI patients might be more accepted. A survey of transfusion preferences of physician leaders at 187 Level 1 trauma centers across the United States found the hemoglobin threshold for transfusion in severe TBI patients varied such that it was 8.3 g/dL for neurosurgeons, 7.5 g/dL for trauma surgeons, and 7.5 g/dL for nonsurgeon intensivists [19]. Salim et al. reported that 76% of anemic (hemoglobin level < 9.0 g/dL) and 21% of nonanemic patients received blood transfusion in the first week of hospital stay [7]. In our study, 40% of patients who had hemoglobin < 10.0 g/dL received PRBC transfusion. The threshold for blood transfusion was frequently higher than 7.0 g/dL, which may be attributed to the presence of shock and the attempt to improve oxygen delivery and reduce secondary brain insult.

The literature shows conflicting findings on the relationship between anemia and outcomes of TBI patients. One study found that a single hemoglobin < 8 g/dL did not predict adverse outcomes such as Glasgow Outcome Scale at 6 months and in-hospital mortality [6]. Another study showed that more days with hematocrit < 30% were associated with improved neurologic outcomes [8]. The current study found that patients who developed anemia (hemoglobin < 10.0 g/dL) during ICU stay had higher mortality (25% versus 6% for nonanemic patients, *p* = 0.01). However, the multivariate analysis showed that anemia was not an independent mortality predictor (OR: 2.5; 95% CI: 0.4–15.8). This could be explained by the fact that the brain has protective mechanisms against anemia and decreased cerebral oxygenation by inducing cerebral vasodilatation and thus maintaining cerebral oxygen homeostasis [20].

In our study, blood transfusion was associated with higher mortality (OR: 6.8; 95% CI: 1.1–42.3). Traditionally, anemia treatment in the critically ill patients, including TBI patients, was liberal blood transfusion to keep hemoglobin level > 10.0 g/dL. However, multiple studies have questioned this practice and showed that blood transfusion was linked with higher mortality and morbidity [15, 21]. In a randomized-controlled trial in critically ill patients, Hébert et al. found that restrictive PRBC transfusion (for hemoglobin < 7.0 g/dL) was associated with 18.7% 30-day mortality which was similar to the mortality (23.3%) associated with liberal transfusion (for hemoglobin < 10.0 g/dL) (*p* = 0.11) [15]. In TBI patients, transfusion was shown to increase cerebral oxygenation but without improvement in cerebral metabolism [22]. However, this may not be attained in all patients. In a study of 60 hemodynamically stable severe TBI patients with pretransfusion hemoglobin < 10.0 g/dL, blood transfusion was associated with an increase in the partial pressure of oxygen in brain tissue in only 78% of patients [23]. Many clinical studies did not show a benefit from blood transfusion in TBI patients. A subgroup analysis of Hébert et al.'s trial evaluated patients with moderate to severe closed head injury following polytrauma and found that the 30-day mortality in the restrictive group was 17% as compared with 13% in the liberal group (risk difference 4.1%; 95% CI: 13.4–21.5%, *p* = 0.64) [9]. In a retrospective review of all blunt trauma patients with TBI admitted to the ICU from 1998 to 2005, blood transfusion was significantly associated with higher mortality (adjusted OR: 2.19; 95% CI: 1.27–3.75) [7]. George et al. found that there were no improved outcomes from transfusing RBC, but there were more complications such as deep vein thrombosis [10]. Carlson et al. found that blood transfusion was associated with poor short term outcomes in TBI patients [8]. Hence, the available studies favor conservative rather than liberal transfusion practices. It is thought that blood transfusion may lead to tissue hypoxia [24], accumulation of potentially toxic microparticles [25], and immunomodulation [26], all of which can lead to organ dysfunction and poor outcome. Another explanation for the worse outcome associated with transfusion could be that PRBC transfusion was given because of dilutional anemia from fluid administration and overload, which by itself could be detrimental [27]. Indication bias, where sicker patients were transfused, may also explain the higher mortality. Human recombinant erythropoietin has been suggested to reduce blood transfusion in ICU patients. A recent trial found that neither treatment with erythropoietin nor maintaining hemoglobin concentration > 10 g/dL by RBC transfusion improved neurological outcome at 6 months [28].

The findings of our study should be interpreted in light of its strengths and limitations. One of its strengths is that we included only patients with isolated head injury. Having other injuries may contribute to anemia and may necessitate blood transfusion in the event of active bleeding and so it will be difficult to evaluate the relationships between anemia, transfusion, and outcomes of TBI. The limitations include the retrospective study design and the lack of data on daily fluid balance and the age of RBCs, which has been suggested to affect the outcome of patients [4]. Moreover, the neurologic outcomes of TBI patients were not evaluated.

In conclusion, anemia became very common during the first week of ICU stay in patients with isolated TBI. PRBC transfusion was frequently used in the management of anemia, even for patients with hemoglobin > 7.0 g/dL. PRBC transfusion, but not anemia, was an independent predictor of mortality. Liberal PRBC transfusion should be avoided in patients with isolated TBI.

## Figures and Tables

**Table 1:** Characteristics of patients according to the occurrence of anemia (nadir hemoglobin less than 10 g/dL) during stay in the intensive care unit and packed red blood cell transfusion status.

	All patients *N* = 101	Anemia during stay *N* = 48	No anemia during stay *N* = 53	*p* value	PRBC transfusion *N* = 21	No PRBC transfusion *N* = 80	*p* value
Age (years), mean ± SD	29.0 ± 14.4	32.1 ± 16.3	26.3 ± 11.9	0.04	30.8 ± 14.8	28.6 ± 14.3	0.54
Male gender, *N* (%)	96 (95)	44 (91.7)	52 (98.1)	0.19	21 (100)	75 (93.8)	0.58
Body mass index (Kg/m^2^), mean ± SD	24.1 ± 4.9	23.4 ± 3.8	24.6 ± 5.6	0.25	23.3 ± 3.5	24.3 ± 5.2	0.42
Preexisting diabetes, *N* (%)	4 (4)	1 (2.1)	3 (5.7)	0.42	0 (0)	4 (5.0)	0.58
Preexisting hypertension, *N* (%)	6 (5.9)	4 (8.3)	2 (3.8)	1.000	1 (4.8)	5 (6.3)	1.0
GCS score on admission, mean ± SD	6.8 ± 3.5	6.3 ± 3.1	7.3 ± 3.7	0.15	6.0 ± 3.2	7.0 ± 3.5	0.25
Patients with GCS score ≤ 8, *N* (%)	81 (80.2)	41 (85.4)	40 (75.5)	0.21	17 (81.0)	64 (80.0)	1.0
APACHE II, mean ± SD	17.9 ± 6.4	18.2 ± 5.0	17.6 ± 7.4	0.70	18.6 ± 5.0	17.7 ± 6.7	0.60
Injury Severity Score, mean ± SD	20.3 ± 7.2	22.3 ± 6.5	18.5 ± 7.4	0.01	22.0 ± 6.8	19.9 ± 7.3	0.25
Type of brain injury^†^, *N* (%)							
Subdural hematoma	25 (24.8)	19 (39.6)	6 (11.3)	0.001	11 (52.4)	14 (17.5)	0.001
Epidural hematoma	21 (20.8)	11 (22.9)	10 (18.9)	0.62	4 (19.0)	17 (21.3)	1.0
Subarachnoid haemorrhage	28 (27.7)	12 (25.0)	16 (30.2)	0.56	2 (9.5)	26 (32.5)	0.04
Intracerebral haemorrhage	18 (17.8)	9 (18.8)	9 (17.0)	0.82	6 (28.6)	12 (15.0)	0.20
Intraventricular haemorrhage	13 (12.9)	4 (8.3)	9 (17.0)	0.20	1 (4.8)	12 (15.0)	0.29
Brain contusion	19 (18.8)	5 (10.4)	14 (26.4)	0.04	2 (9.5)	17 (21.3)	0.35
Diffuse axonal injury	9 (8.9)	2 (4.2)	7 (13.2)	0.16	2 (9.5)	7 (8.8)	1.0
Hemoglobin on admission (g/dL), mean ± SD	14.1 ± 2.1	13.4 ± 1.9	14.7 ± 1.9	0.002	13.0 ± 2.1	14.4 ± 1.9	0.004
PaO_2_ : FiO_2_^‡^, mean ± SD	316 ± 107	336 ± 107	297 ± 104	0.13	364 ± 117	306 ± 103	0.08
MAP on day 1^**∗**^ (mmHg), mean ± SD	91 ± 8	91 ± 8	89 ± 8	0.58	95 ± 5	88 ± 8	0.04
ICP on day 1^**∗**^ (mmHg), mean ± SD	17 ± 11	19 ± 12	14 ± 8	0.41	23 ± 14	14 ± 7	0.09
CPP on day 1^**∗**^ (mmHg), mean ± SD	73 ± 10	73 ± 11	75 ± 7	0.63	71 ± 13	74 ± 8	0.47
Management in the intensive care unit, *N* (%)							
Mechanical ventilation	98 (97.0)	48 (100.0)	50 (94.3)	0.24	21 (100)	77 (96.3)	1.0
Head injury protocol	76 (75.2)	42 (87.5)	34 (64.2)	0.01	19 (90.5)	57 (71.3)	0.07
Craniotomy	31 (30.7)	24 (50.0)	7 (13.2)	<0.001	9 (42.9)	22 (27.5)	0.17
ICP management	32 (31.7)	21 (43.8)	11 (20.8)	0.01	11 (52.4)	21 (26.3)	0.02
PRBC transfusion	21 (20.8)	19 (39.6)	2 (3.8)	<0.001	21 (100)	0 (0)	0.0001
Other transfusions^*∗∗*^	15 (14.9)	14 (29.2)	1 (1.9)	0.0001	8 (38.1)	7 (8.8)	0.002

APACHE: Acute Physiology and Chronic Health Evaluation; CPP: cerebral perfusion pressure; GCS: Glasgow Coma Scale; ICP: intracranial pressure; MAP: mean arterial pressure; PaO_2_/FiO_2_: the ratio of partial pressure of oxygen to the fraction of inspired oxygen; PRBC: packed red blood cell; SD: standard deviation.

^†^One patient may have more than one type of brain injury. For example, 9 patients with subdural hematoma had other injuries.

^‡^Missing data for 31 patients.

^*∗*^Applies to patients who had intracranial pressure monitoring; only one patient who did not develop anemia during stay had intracranial pressure monitoring.

^*∗∗*^Platelets and/or fresh frozen plasma.

**Table 2:** Outcomes of patients according to the occurrence of anemia (nadir hemoglobin less than 10 g/dL) during stay in the intensive care unit and PRBC transfusion status.

	All patients *N* = 101	Anemia during stay *N* = 48	No anemia during stay *N* = 53	*p* value	PRBC transfusion *N* = 21	No PRBC transfusion *N* = 80	*p* value
Seizures, *N* (%)	12 (11.9)	3 (6.3)	9 (17.0)	0.10	1 (4.8)	11 (13.8)	0.45
Ventilator-associated pneumonia, *N* (%)	18 (17.8)	8 (16.7)	10 (18.9)	0.77	3 (14.3)	15 (18.8)	0.76
Venous thromboembolism, *N* (%)	5 (5.0)	4 (7.6)	1 (1.9)	0.19	0 (0)	5 (6.3)	0.58
Tracheostomy, *N* (%)	37 (36.6)	20 (41.7)	17 (32.1)	0.32	9 (42.9)	28 (35.0)	0.45
Duration of mechanical ventilation (days), mean ± SD	7.9 ± 5.4	8.8 ± 5.1	7.1 ± 5.7	0.13	9.3 ± 5.5	7.6 ± 5.4	0.19
ICU LOS (days), mean ± SD	9.6 ± 7.1	9.2 ± 8.0	10.1 ± 5.8	0.55	9.8 ± 6.5	9.6 ± 7.2	0.92
Hospital LOS (days), mean ± SD	59.8 ± 79.9	68.6 ± 94.1	51.8 ± 64.4	0.30	82.0 ± 100.1	54.0 ± 73.4	0.24
ICU mortality, *N* (%)	14 (13.9)	11 (22.9)	3 (5.7)	0.01	8 (38.1)	6 (7.5)	0.001
Hospital mortality, *N* (%)	15 (14.9)	12 (25.0)	3 (5.7)	0.01	8 (38.1)	7 (8.8)	0.003

ICP: intracerebral pressure; ICU: intensive care unit; LOS: length of stay; SD: standard deviation.
